# Recent Developments in the Mechanical Behavior of Polymer-Based Composites

**DOI:** 10.3390/polym18050598

**Published:** 2026-02-28

**Authors:** Marcelo Antunes, David Arencón

**Affiliations:** Poly2 Group, Department of Materials Science and Engineering, ESEIAAT, Technical University of Catalonia (UPC BarcelonaTech), C/Colom 11, 08222 Terrassa, Spain; david.arencon@upc.edu

**Keywords:** mechanical behavior, polymer composites, nanocomposites, nanohybrids, multiscale reinforcements, bio-based polymer composites, smart materials, advanced additive manufacturing, artificial intelligence, machine learning

## Abstract

Polymer-based systems have been shown to have a particular combination of characteristics that make them desirable in technological sectors, such as lightness, insulating properties, and easy molding during processing, as well as mechanical versatility, which is greatly due to their molecular microstructure. Nevertheless, they still present limitations in mechanical performance and use at moderate/high temperatures, considerably restricting their range of applications. Thus, great efforts have been directed towards developing strategies intended to enhance said characteristics and predict their complex mechanical behavior, with the main goal of adapting their properties to the end-use application. The present review considers the most recent developments, focusing on the research published in 2025 and early 2026, and future challenges in the mechanical behavior of polymer-based materials, being structured according to material considerations, more specifically the development of advanced (nano)composites based on high-performance matrices and functional nanoparticles, as well as bio-based polymer (nano)composites obtained from renewable sources and multifunctional smart and meta-materials for monitoring and long-term use; the development of new processing methods, focusing on advanced additive manufacturing; and the use of artificial intelligence and machine learning. All in all, the final objective is generating knowledge that will enable the preparation of components with tailor-made mechanical characteristics and functional properties, covering material design and processing.

## 1. Introduction

Polymers are a group of materials that, given their particular molecular microstructure and aggregation state (amorphous or semi-crystalline), display a vast range of mechanical characteristics, in many cases being non-linear due to viscoelastic behavior and highly dependent on the applied external mechanical stimulus (static load or fatigue; type: tensile, compressive, flexural, etc.), on how it is applied (low velocity or impact conditions), and on the external conditions (use temperature, occasional or sustained over time, humidity, and exposure to chemicals, among others). As a result, polymers may display an overall mechanical behavior that can be brittle (for instance, cured thermosets or thermoplastics below their glass transition temperature), more or less ductile, commonly with considerable plastic deformation (for instance, semi-crystalline thermoplastics above their glass transition temperature and below their softening point), or even elastomeric, with significant recoverable elastic deformation even at low values of applied stress (characteristic of elastomers). This high mechanical versatility, albeit in some cases difficult to analyze and predict, together with their lightness and insulating properties, as well as easier molding when compared to other materials, explains the great boost in recent years of these materials in both research and industrial worlds. Great efforts have been made in predicting their mechanical behavior, with the final objective of tailoring their characteristics according to the end-use application, by adjusting the molecular structure (length of the molecules, molecular architecture, copolymerization, etc.); by controlling their possible degree of crystallization; by combining them with additives (like plasticizers or impact modifiers), especially with different types of reinforcements (polymer composites), from common glass or carbon fibers to more recent nanosized reinforcements (polymer nanocomposites) like carbon nanotubes or graphene; and by rationally selecting the processing technology and conditions.

As far as we know, there are not any prior similar reviews that, to begin with, deal with such recent and ongoing research and specifically cover all the aspects considered in the present review, i.e., how material considerations (with special focus on profound aspects of multiphase material design), the proper selection of the processing method (namely the suitability of already existing trending technologies and the expected development of new ones specifically adapted to each multiphase material), and the use of AI and potent ML methods to predict the short-, mid- and long-term complex mechanical performances of these materials, which can make feasible the design of components with tailor-made mechanical and functional properties for a specific application.

This review covers the most recent developments in the mechanical behavior of polymer-based materials, especially polymer (nano)composites, focusing mainly on the research work published in 2025, early 2026, and ongoing, dealing with the three main aspects governing the mechanical performance of said materials (see Table 1): (1) material considerations, more specifically the development of novel advanced composites and nanocomposites based on high-performance matrices and functional nanoparticles (including nanohybrids and multiscale reinforcements) and, given the increasing awareness in developing eco-friendly and sustainable materials, bio-based polymer (nano)composites obtained from renewable sources, as well as multifunctional smart materials and metamaterials for monitoring and long-term extension; (2) the development of new processing methods and technologies, focusing on advanced additive manufacturing (3D/4D printing and hybrid methods); and (3) the use of artificial intelligence (AI) and machine learning (ML) for characterizing complex mechanical behaviors, inherent to such multiphase systems, boosting material development, all in all with the main goal of generating knowledge that will enable the preparation of parts with tailor-made mechanical characteristics and functional properties, covering both material design (composition and microstructure) and processing (selection of the technology according to the required characteristics of the part), favoring an application-driven type of approach (see Table 2).

## 2. Advanced Composites and Nanomaterials

When dealing with the recent trends in the mechanical performance of polymer-based materials/components for structural engineering applications, perhaps the first and more obvious one deals with the development of novel polymer-based formulations with enhanced mechanical properties through a rational combination of components, from the use of high-temperature and highly resistant polymers (thermosets or thermoplastics, with special interest in the second type), polymer blends/mixes, the creation of new polymer-based composites, with special interest being given to the addition to polymer-based matrices of functional nanosized fillers (nanocomposites), alone or combined (nanohybrids) or even combining multiscale fillers, to the development of lightweight polymer-based foams.

### 2.1. Advanced Composites Based on High-Performance/High-Temperature Polymers and Blends

A recent trend in terms of material development deals with advanced composites based on high-performance/high-temperature polymers, with special interest in the development of novel thermoplastic-based formulations, as traditionally thermoset-based composites have been more commonly used. Primarily polyimides reinforced with carbon fibers, which can maintain mechanical integrity at temperatures as high as 350–400 °C, are often processed by prepreg consolidation using compression molding or inside autoclaves [1,2]. High-temperature thermoplastics do not withstand such high temperatures. However, they often show superior toughness and chemical resistance than thermosets, besides obvious advantages in recyclability. This has aroused great interest in the composites world, particularly in sectors where their versatile and high-performance characteristics under extreme conditions may be used to their fullest potential, such as aerospace or automotive. The most considered thermoplastics have been polyetheretherketone (PEEK), polyetherimide (PEI), and polyarylsulfones (polysulfone, PSU; polyethersulfone, PES; and polyphenylsulfone, PPSU) [3,4]. AM-based processing methods have been recently considered, given the significant recent improvements in AM accuracy and reliability [5,6], though testing is limited to the short term, still raising issues about the long-term viability of 3D printed parts. The integration of AI and ML methods is expected to speed up the development of novel materials and improve the performance of already existing ones, adapting them better to the selected processing technology. Sustainability will continue to be a significant motivation, encouraging the development of novel bio-based polymer systems with enhanced mechanical and thermal stabilities.

**Figure 1:**
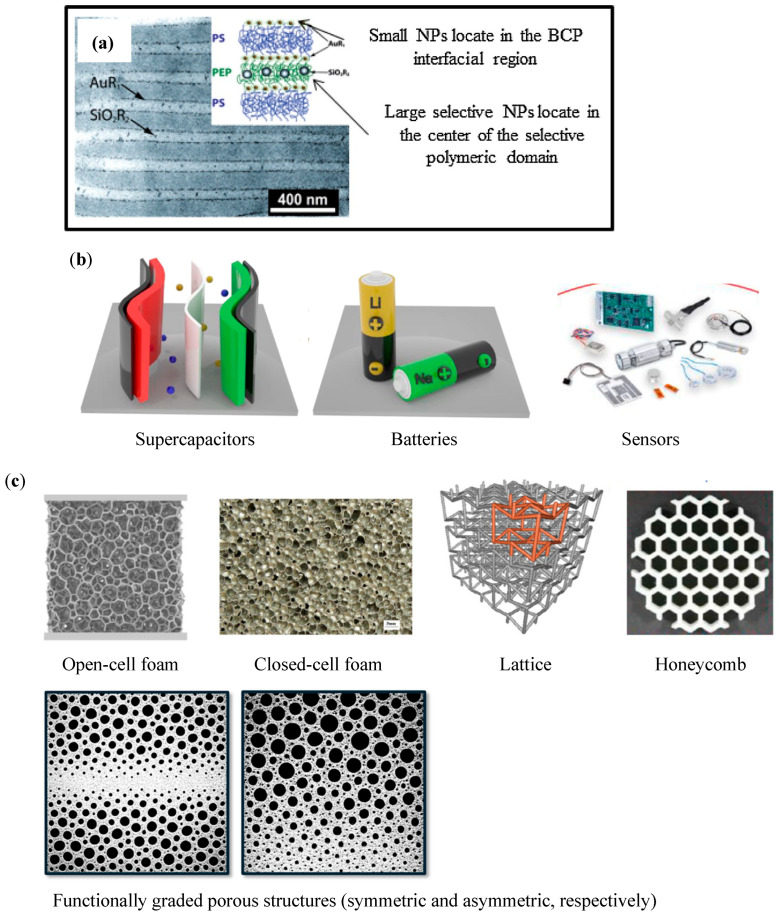
(**a**) Influence of the size of NPs on their selective localization in block copolymers (adapted from [10]. Elsevier, 2015); (**b**) possible applications of polymers reinforced with nanohybrids; and (**c**) examples of controlled-porous structures (adapted from [86]. Elsevier, 2023; functionally graded porous structures adapted from [98]. Elsevier, 2026).

Singh et al. [7] have recently reviewed the interesting possibilities of using block copolymers (BCPs) as materials that have the ability to self-assemble into controlled microdomains, which, in combination with the addition of nanosized fillers, could lead to the development of next-generation functional hybrid materials with enhanced mechanical performance, alongside additional characteristics such as electrical or thermal conductivities. In particular, it is demonstrated that the particular molecular organization of block copolymers enables a precise control of the localization of the nanoparticles and their distribution within its structure, facilitating tailoring of the material’s properties (see example in Figure 1a). As proper nanoparticle placement depends considerably on ensuring a strong interaction between the nanoparticle surface and the polymer matrix, great efforts have been directed toward surface modifying the nanoparticles [8]. This makes them more compatible with the matrix. In the particular case of block copolymers, they can even be made selectively compatible with only one or both polymer blocks [9,10,11]. This facilitates localized nanoparticle dispersion in only the compatible block or in the interface region between blocks when they are selective to both. Although significant developments have been made in this field, there are still some issues on how to properly assess the dynamic real-time assembly behavior of these complex systems, which is crucial for defining their properties. Also, as many industrial applications consider the use of these materials as thin films coated on the surface of substrates, since it is known that in these cases the self-assembly of BCPs is influenced by additional factors, there is still the need to study and generate more data on BCP hybrids containing nanoparticles in thin films.

### 2.2. Advanced Polymer-Based Nanocomposites

Significant work has been conducted in the last decade and is still ongoing on the addition of nanosized reinforcements to polymer-based systems for mechanical and overall functional enhancement. This ranges from the initial silicate-layered nanoclays to functional carbon-based nanofillers, namely different types of carbon nanotubes and/or graphene-based materials [12,13,14,15,16]. These have even been considered as the possible building blocks for the future [17] or, even more recently, the use of two-dimensional MXenes [18,19]. As with composites, a great deal of recent developments in these materials have been application driven. Ongoing research has focused on sensors and actuators, which are smart materials for deformation, temperature, gas, and biosensing [20,21]. This is due to the possible tailor-made response of these materials to external stimuli. Research has also emphasized energy storage, such as lithium-ion and metal–air batteries [22,23]. Here, carbon-based nanomaterials may act as both active electrodes and electrically conductive additives, enhancing the efficiency and performance of the battery. Additionally, studies have targeted supercapacitors: for instance, layered graphene-based materials, given their unique combination of properties, are expected to find application in flexible supercapacitors.

As with composites in general, there are still significant challenges that need to be properly addressed if polymer-based nanocomposites are to be used at an industrial scale, namely material availability (high volume synthesis and, in the particular case of nanocomposites, difficulties in guaranteeing a homogeneous distribution and dispersion of the nanoparticles throughout the matrix), cost effectiveness, and overall long-term mechanical and thermal stabilities, with similar challenges in terms of improving material recyclability through the use of bio-based polymers and natural/biodegradable fibers (biocomposites [24,25,26]) or the development of AI and ML methods for assisting in material design.

The combination of different functional nanosized fillers, which commonly have different geometries, has been explored. For instance, tubular-like carbon nanotubes can be combined with layered graphene-based materials (such as graphene nanoplatelets, graphene oxide, reduced graphene oxide, and, more recently, graphene nanoribbons). These are usually named “nanohybrids” [27,28,29]. They have been proven to show synergies when added into polymer-based systems, leading to enhancements in mechanical performance and transport properties (multifunctionality), such as electrical conductivity or EMI shielding efficiency. This opens new possibilities in electronic components (batteries, supercapacitors, sensors, and actuators; see Figure 1b) [30,31,32,33,34]. For instance, Shchegolkov et al. [31] have shown that the combination of CNTs and graphene nanoplatelets, given the conditions (proper dispersion throughout the polymer matrix and enhanced interface strength promoted by prior nanoparticle’s surface modification [35,36,37,38], as well as proper nanohybrid structure control through processing [39,40,41]), promotes the formation of a hybrid connected 3D network in polymer-based systems, enabling the control of strength, stiffness, and electrical conductivity of nanocomposites, ultimately permitting the development of micro/macro-deformation-detecting sensors for structural monitoring.

Also interesting is the possibility of maximizing the efficiency of common microscopic reinforcements, namely carbon fiber, by combining them with functional nanoreinforcements (CNTs and/or GnPs), giving way to what are known as “multiscale reinforcements”. It has been shown that some of the limitations of carbon fiber-reinforced polymers, such as the weak interface that tends to form between fibers and the matrix, may be counteracted by combining said fibers with low amounts of CNTs and/or GnPs, including graphene-based materials and carbon nanotube chemical, physical, and chemical–physical functionalization strategies [42,43,44,45,46,47]. Recent works seem to point in the direction of developing selective functionalization methods to effectively control the location and density of functional groups, optimizing interface properties and counteracting one of the main limitations of nanoparticle functionalization, which is the negative impact of functional groups on the intrinsic properties of the nanoparticles [48]. Other works have focused on the use of environmentally friendly functionalization approaches [49] and the development of automated systems with real-time monitoring for tailor-made nanoparticle modification and incorporation into composites [50]. As can be seen, future trends will focus on developing complex multiscale-reinforced systems, the use and/or integration of advanced 3D/4D printing processes, and the utilization of AI and ML.

Among nanofillers, besides carbon nanotubes and graphene-based materials, there has been an increasing interest in the use of nanocellulose as a reinforcement [51,52,53], as cellulose nanocrystals (CNCs), nanofibrils (CNFs), and bacterial nanocellulose (BNCs) have been shown to be a viable reinforcement for polymer-based systems [54], especially bio-based, and in recent years, new processes have enabled a faster production rate, a reduced environmental impact in terms of nanocellulose production (for instance, using new fibrillation techniques [55,56]), and, as a result, a greater material availability and cost reduction. Also, big developments have been made in the chemical modification and functionalization of nanocellulose, enhancing the compatibility with a higher number of polymer matrices [52,57,58,59,60]. Nanocellulose has also been recently combined with other nanofillers, such as carbon nanotubes [61,62,63] and MXenes [64], in this last case combining the mechanical strength and high aspect ratio of bio-based nanocellulose with the high electrical conductivity and surface area of platelet-like MXenes, taking advantage of the strong hydrophilic surface interactions between both [65]. Nanocellulose/MXene-reinforced polymer composites have shown possibilities as EMI shielding components [66,67,68], supercapacitors, sensors [69,70], and in water treatment applications.

As generic limitations to the extensive use of nanocellulose include scalability, reusability, and long-term durability, strategies to overcome these issues have considered the development of advanced composites and hybrid materials, combining nanocellulose with other functional nanofillers, the need for continued optimization of new processing techniques, and the development of new smart nanocellulose-based systems.

### 2.3. Porous Polymer-Based Structures

Another interesting aspect when dealing with recent/future developments of polymer-based systems with enhanced mechanical performance deals with the simultaneous creation of a controlled porous structure, especially considering the interest in further reducing the global weigh of the final component (a crucial aspect in aerospace or automotive sectors), enhancing some of the functional properties of the material (for instance, reducing thermal conductivity for insulating purposes), or even promoting synergies between foaming and the addition of nanosized fillers, which could ultimately result in advanced lightweight components with improved specific properties for structural purposes. In this sense, polymer-based porous structures, including polymer foams, have evolved from their conventional applications, such as passive insulating components, to multifunctional materials that combine enhanced mechanical performance and durability with functional active characteristics, such as sensing or self-repair [71]. Future research seems to point in the direction of bio-inspired cellular designs, from foams to lattices or honeycombs mimicking naturally occurring hierarchical structures that have been proven to work from a mechanical point of view. Together with cellular/porous structure control, novel bio-based formulations have also been considered [72,73,74,75], in some cases combining bio-based polymer matrices with lignocellulosic reinforcements [76,77,78,79]. Besides bio-based formulations, there is great interest in further developing and extending the knowledge of novel porous structures/foams created from polymer-based nanocomposites. This is especially true for nanocomposites that incorporate functional nanosized fillers, more specifically carbon-based ones (such as carbon nanotubes, graphene-based materials, nanocellulose, etc.) or MXenes [80,81,82,83,84,85]. The synergies between low amounts of these functional nanoparticles and the generation of a controlled cellular/porous structure could result in lightweight materials with non-conventional characteristics. These include electrical conductivity and improved EMI shielding efficiency.

Given the range of novel applications that foams/porous materials may open in several sectors—especially considering the increasingly demanding required use conditions—researchers are addressing the development of functionally graded porous structures. These are commonly divided into lattices, honeycombs, and foams. They adapt the characteristics of the component to its use requirements based on a controlled graded structure (see examples of controlled porous structures in Figure 1c). This adaptation can involve the cellular structure, composition, or a combination of the two [86]. However, changes in cellular morphologies tend to be easier to attain when compared to the necessity of selectively controlling the material’s composition. Combination of both strategies has been considered [87]. This involves forming a porous structure from composites previously prepared by combining a polymer-based matrix with nanofillers, namely carbon-based ones such as carbon nanotubes and/or graphene-based materials. It has been shown that foaming may promote a better distribution/dispersion of the nanofillers or their selective localization throughout the cell walls, hence enhancing the functional efficiency of said nanofillers [88,89,90]. Additionally, the presence of the nanoparticles, besides favoring the formation of finer cellular structures, may mechanically reinforce the materials and add additional functional characteristics.

As porous structures include lattices and honeycombs, recent fast developments in additive manufacturing have enabled easier and fast preparation of components with desired graded cellular structures [91,92,93,94,95,96,97]. Additive manufacturing will be dealt with in more detail in a later section, including recently developed AM-based methods that could solve some of the current common problems of using AM to create graded porous structures, which include rheological considerations of the material during printing, control of the porous structure being generated, or a lack of standardized methods to characterize porosity. Emerging technologies include ML-based processes; the development of novel printable materials, especially in the case of filament-based printing methods; and the development of advanced hybrid-like printing processes.

Future directions regarding functionally graded porous components with enhanced specific mechanical properties point to creating design guidelines that incorporate porosity gradients and new types of materials, preferably sustainable; the use of AI-assisted design and ML by progressively incorporating experimental data; creating solid relations between graded porosities and material properties; developing multiscale computational tools to assess the influence of the porous structure on the properties; and developing new large-scale processing methods, such as advanced additive manufacturing, making possible the mass production of these multiphase lightweight materials [98].

### 2.4. Interface Engineering

**Figure 2:**
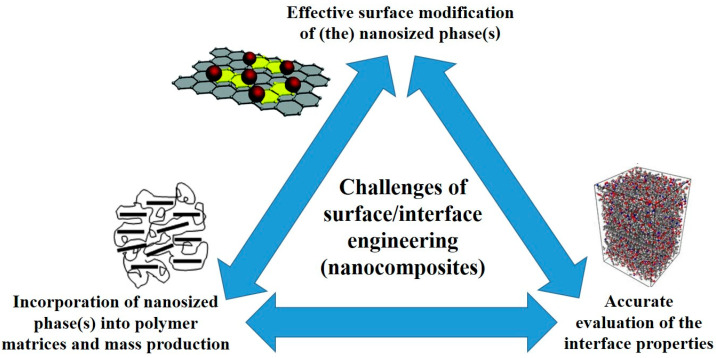
Challenges of surface/interface engineering of polymer-based nanocomposites.

As mentioned before, many of the strategies behind the development of composites with enhanced mechanical performance consider the use of nanoreinforcements. These can be used alone or combined (nanohybrids), either in the same scale or multiscalar: for instance, combining nanoscale reinforcements with more conventional microscopic ones, especially fibers such as carbon or glass fibers. The “quality” of the interface established between the matrix and said nanoreinforcements gains in importance. This interface is considerably higher in volume when compared with more conventional reinforcements, due to the high specific surface area of the nanofillers. As a result, in conventional polymer-based composites, reinforcements are typically fibers, commonly continuous. Here, the interface between the matrix and said fibers is low in volume when compared to the volume fraction of the individual components (matrix and reinforcement). Hence, in many cases, it can be disregarded when predicting their mechanical performance. In contrast, in advanced composites with nanoreinforcements, it is quite critical to control said interface. This is because it may significantly affect the final mechanical properties of the composite and, in many cases, will mark the possible advantages in terms of mechanical enhancement between these advanced composites and the more conventional ones. In this sense, when dealing with the strategies being used to enhance the mechanical performance of composites through material selection and preparation, one of the most recent trends is interface engineering. This involves enhancing the “quality” of the interface between nanoreinforcements and polymer matrices. Specifically, it focuses on improving the compatibility and adhesion between these two phases and controlling the molecular characteristics of the matrix, in some cases even inducing specific molecular orientations. Overall, the objective is to enhance the strength of the resulting composites. More specifically, focus has been placed on identifying, predicting, and controlling the correlations between the interface(s) across the different possible materials’ scales (nano, micro, and macro) and the multifunctional properties of the resulting composites [99,100,101]. In the specific case of nanosized reinforcements, especially those with intrinsically high mechanical and transport properties such as carbon-based nanoreinforcements (carbon nanotubes and graphene-based materials, among others [102,103,104]), surface and interface engineering may come as a relatively cost-effective and quick strategy to achieve multifunctionality when compared to atomistic/molecular approaches (bottom-up; structure/morphology engineering) [105]. Surface engineering deals with the modifications on the surface of nanoreinforcements, including chemical structure and morphology, adjusting the interface interactions between said nanoreinforcements and the polymer matrix, and enabling further optimization of the composites’ performance [106,107]. For instance, carbon-based nanoreinforcements can be surface modified through the introduction of functional groups, strengthening the interaction with the polymer matrix, both at a physical as well as a chemical level [108]. Even though great advancements have been achieved in the last decade in adjusting and controlling the surface and interface engineering of nanosized reinforcements, maximizing their mechanical and functional efficiencies when added to polymer matrices, and trying to fulfill the increasingly higher stringent requirements for polymer-based components in the most varied fields (automotive, aerospace, electronics, etc.), there are still some important non-fully resolved challenges. These can be divided in three main aspects (Figure 2): (1) how to effectively surface modify the nanosized phase(s), as there are still issues of surface modifications negatively affecting the intrinsic original properties of the nanoreinforcements [109,110,111,112]; (2) how to properly incorporate the surface-modified nanoreinforcements into the polymer matrix and scale it up to high-volume processes, as common surface/interface engineering methods such as laser processing are too expensive and chemical functionalization may require high-cost, toxic reagents and quite stringent reaction conditions [99]; and (3) how to accurately evaluate the interface properties in these multiphase systems, as current characterization methods show difficulties in reliable sample preparation and limitations in assessing interfacial properties, leading to significant experimental errors. Though AFM-related techniques such as nanoindentation have recently enabled to assess interface characteristics such as modulus or strength [113,114], testing is still quite slow and dependent on sample preparation, hence the necessity to develop more properly adapted in situ characterization techniques. In order to overcome experimental limitations, taking advantage of recent developments in computer analysis, molecular simulation methods have emerged as possible tools to assess the interfacial properties of composites at micro/nanoscales. Nevertheless, they depend on the use of reliable and accurate data, still significantly missing for such complex multiphase systems. In this sense, novel enhanced machine learning methods might solve this issue [115,116], leading to the design of optimal interfaces.

## 3. Bio-Based Polymer (Nano)Composites

Albeit already quite present for some years in the scientific world, another trending topic regarding the rational selection of materials in polymer-based systems for optimization of mechanical performance and overall functionality and recyclability deals with the use of sustainable and bio-based polymers, namely those obtained from renewable resources rather than petroleum, in some ambits called “green polymers” [117,118,119]. There is an obvious positive environmental impact in using them, as they reduce fossil fuel dependence and carbon dioxide emissions. On the other hand, though used as synonyms in many contexts, not all bio-based polymers are biodegradable, i.e., not all degrade when exposed to specific conditions, namely when in contact with microorganisms or when subjected to aerobic and/or anaerobic processes. Such is the case of some “green polymers” like bio/green polyethylene. In the same way, there are examples of biodegradable polymers that are not bio-based, such as polycaprolactone [119]. Interestingly, common carbohydrate agricultural feedstocks (corn, rice, etc.) have been replaced in recent years as the source of bio-based polymers by polymers synthesized using microorganism fermentation processes from renewable resources such as lignocellulosic or organic biomass (cellulose or nanocellulose [120]). Naturally occurring bio-based polymers (proteins, chitosan, collagen, etc.) have also grown in interest in recent years, both at a technological level as well as commercial. Bio-based polymers can be classified in terms of how they are produced from renewable resources in those that are partially modified from natural bio-based polymers (ex.: thermoplastic starch); those obtained from the polymerization of bio-based monomers produced by fermentation or rather conventional chemistry (examples: polylactic acid and biopolyethylene); and those directly produced by bacteria, such as polyhydroxyalkanoates [117].

**Figure 3:**
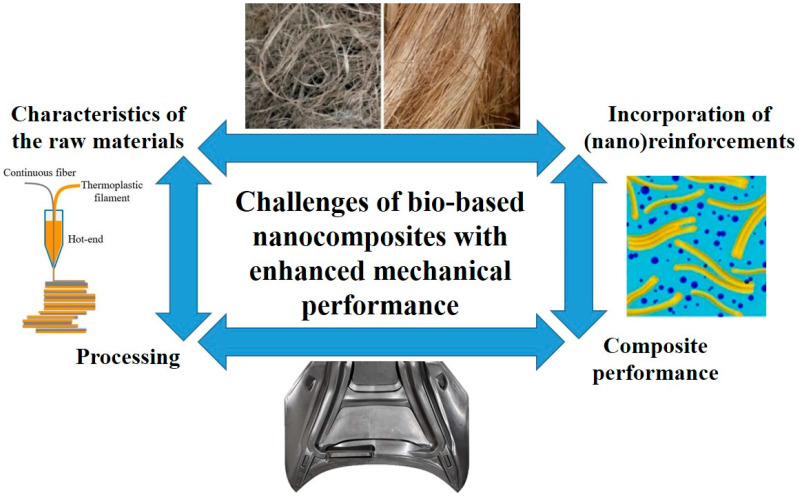
Current and future challenges of bio-based polymer composites with enhanced mechanical performance.

As mentioned before, great importance has been given in recent years to bio-based polymers when developing novel sustainable polymer-based composites with enhanced mechanical performance as an alternative to conventional composites that combine thermoset matrices with synthetic fibers (glass, carbon, or even hybrids) [121,122,123]. More specifically, extensive research has dealt with challenges related to the long-term durability and reliability, as well as the overall thermal and mechanical properties and sustainable production of these materials. Current and future challenges can be summarized in the following points (Figure 3):

(1) Traceability and control of the characteristics of the raw materials, which arise from the inherently heterogeneous nature of biofibers or their surface characteristics mismatch with a great number of polymers, even bio-based [121,124]; exploring the development of new biofibers and polymer resources; and effective production in industrial amounts.
(2) Raw material cost variability, as the price of bio-based materials (polymers and fibers) is highly dependent on cultivation conditions and crop yield [125].
(3) Possible problems when incorporating (nano)reinforcements, especially bio-based ones such as nanocellulose into bio-based polymers (moisture and surface characteristics, among others) and overall performance. Current approaches and advances have focused on surface treatment and modification of the (nano)reinforcements using chemical, physical, and biological methods, with the objective of enhancing their compatibility with the matrix [126,127,128,129,130,131,132,133], and hybridization [134,135,136], including the interesting possibility of multiscalar hybridization taking advantage of functional synergies between (nano)reinforcements [81].
(4) Stability of the resulting composites, especially in terms of their service/use temperature.
(5) Processing, effectively transferring knowledge from the lab to the industry with the development of new manufacturing routes that enable high yields, the extended use of new microorganisms (fermentation processes) [137,138,139], and efficient downstream processes for bio-based product recovery [140,141,142]. Among what are considered as advanced manufacturing techniques, additive manufacturing (AM), which will be dealt with in more detail in a later section, offers great opportunities in the production of sustainable and multifunctional bio-based polymers for a vast array of sectors, from aerospace to packaging [121]. Nevertheless, albeit being an economic and even scalable type of process (though scalability still remains limited [143,144]), enabling for custom geometries and functionalities, it shows limitations in terms of reliability and even reproducibility, especially when dealing with high-performance components. Typical defects include voids or porosity between printed layers or between matrix and fibers and poor surface finish. New AM-based techniques also need to be developed in order to allow for proper preparation of continuous fiber-reinforced thermoplastic-based biocomposites for structural applications, given the current limitations of proper fiber impregnation due to the high melt viscosity of thermoplastics [121,145]. In the case of nanoreinforcements, there are still problems in guaranteeing uniform distribution and dispersion, as most nanoparticles tend to aggregate during processing. In this sense, the prior preparation of new polymer-based feedstocks already containing the required and properly distributed/dispersed amount of nanoparticles could solve this problem. As mentioned before, surface modification of the nanoparticles could facilitate distribution/dispersion and guarantee a better interface bonding with the matrix, leading to improved thermal stability and mechanical performance [146]. Another interesting aspect of AM deals with the possibility of creating multilayer components with multiscalar reinforcements [147].
(6) Widening the application range of bio-based composites.

Since sustainability is not only related to biodegradability or the use of bio-based polymers but also considers broader strategies related to recycling or energy consumption reduction, there has been a gradual shifting, mostly in the scientific world but also starting in the industrial one, in the use of thermoplastics, especially high-performance thermoplastics [148], instead of thermosets as the matrix in polymer composites [118,149]. Thermoplastic-based composites are expected to significantly reduce plastic waste, given their much wider range of possible recycling processes when compared to thermosets, including mechanical recycling, greater possibilities in reuse, easier separation from reinforcements, recently developed novel chemical recycling processes, or upcycling. Thermoplastic composites also display better fatigue resistance, faster forming cycles, and no particular storage condition restrictions (though thermoset shelf life may reach years, some types only last a few months, depending on temperature and humidity storage conditions). Hence, sustainable polymer-based composites with enhanced mechanical properties based on high-performance thermoplastics have been the focus of scientific research in recent years and are expected to continue their development in coming years. Some of the strategies being considered for high-performance applications where strength and mechanical and thermal stabilities are crucial (aerospace, automotive, electronics, etc.) include the (1) development of novel thermoplastic-based prepregs [150,151,152], using different processes such as solution, melt and powder impregnations, as well as film lamination of the fibers for prepreg preparation and later composite production through molding processes (hot pressing, automatic laying, in situ consolidation, or even 3D printing). It has been proven that high-performance composites prepared from said thermoplastic prepregs display improved impact resistance and a proper balance of strength and toughness, in addition to enhanced thermal stability and in some cases improved water resistance (for instance, using 3D printing from previously prepared water-resistant prepreg filaments having a core–shell structure [152]), enabling their use for high-demand engineering applications. (2) Moreover, this includes the recent interest in shape memory thermoplastic composites for medical purposes, actuators, and structural components, with advantages in (re)processing and deformation and recovery ability when compared with thermoset-based ones [153]; and (3) self-healing thermoplastic composites, trying to solve one of the most critical issues when dealing with common thermoset-based ones, which is their susceptibility to micro-crack during use, affecting their overall mechanical performance and in many cases resulting in their swelling due to moisture and even brittle fracture, as well as challenges in terms of repair [154]. Although there are still some challenges when adding self-healing thermoplastics to thermoset-based matrices, as they tend to diminish composites’ overall mechanical strength, they have been shown to enable the early assessment of possible damage, facilitating repair and increasing the service life of components. Additional challenges are related to how to transfer and integrate all these possibilities into practical applications under various environmental conditions [155].

## 4. Multifunctional Polymer-Based Materials

**Figure 4:**
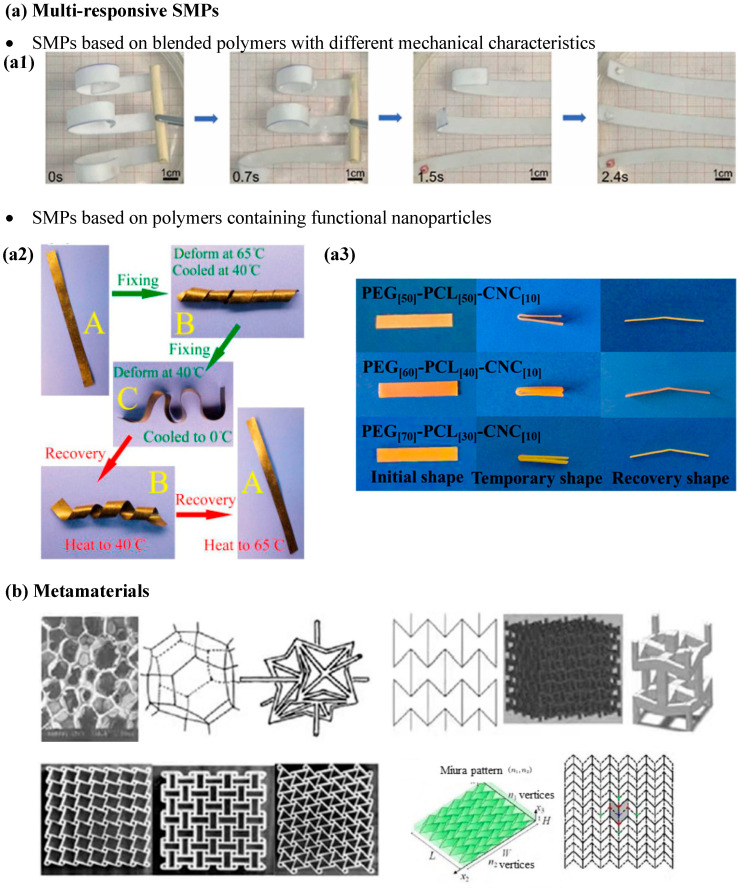
(**a**) Multi-responsive shape memory polymer-based materials (SMPs): (**a1**) PLA/TPU/HA shape memory blend (adapted from Ye et al. [159]. Elsevier, 2025), (**a2**) SMCs containing CNTs (adapted from Dong et al. [172]. Elsevier, 2015), and (**a3**) SMCs containing cellulose nanocrystals (adapted with permission from Liu et al. [173]. American Chemical Society, 2015); (**b**) examples of metamaterials (adapted with permission from Gu et al. [168]. Elsevier, 2025).

Besides the previously mentioned strategies, recent research has focused on lifespan extension and monitoring through multifunctionality (use of smart materials [156,157]) and the development of polymer-based metamaterials [158]. To some extent, these strategies combine the development of novel materials by adjusting material formulation/composition through a rational combination of different materials. They also involve the control of the interface characteristics between secondary phases and polymer matrices, as well as how proper selection of the processing method may induce specific microstructures in the resulting composites. The ultimate goal is to enhance mechanical performance and thermal properties. Particular technological interest has been given to multi-responsive/multifunctional shape memory polymer-based materials (SMPs), mentioned already in a prior section, especially those that blend polymers with different mechanical characteristics [159,160,161] and those containing functional nanoparticles (carbon-based nanofillers [162] and nanocellulose, among others) for improved mechanical performance (hence extending the range of possibilities to structural), the combination of which resulting in what are known as “smart materials” (see Figure 4a). Multifunctional shape memory polymer nanocomposites are the consequence of their multi-responsive behavior, where their shape, i.e., deformation, may be modified in a controlled way upon applying changes to different external stimuli, from temperature to relative humidity or electric/magnetic fields [163]. In this sense, novel materials with the ability to memorize more than one shape and reversible mechanical recovery have been recently developed and have begun to be addressed. Also, new ways of promoting shape modification in common thermally triggered SMPs are being considered, namely using indirect Joule effect heating or alternative stimuli such as chemical reaction energy or changes in externally applied magnetic fields [157,164].

Similar to smart materials and owing to their periodic microstructure [165,166,167], metamaterials display high mechanical performance and programmable deformation, enabling their recent consideration as structural substrates for the integration of functional smart materials for applications such as active sensing [158], as well as for the development of energy absorption, noise reduction, or thermally conductive materials [168]. However, the application of mechanical metamaterials—from auxetic or lattice metamaterials to metamaterials with negative stiffness or Origami-like [168] (see Figure 4b)—in multifunctional devices has not fulfilled its initial technological expectations. This is due to the processing technologies that still lack the required precision to fully attain the expected performance based on initial design. However, its industrialization has been accelerated in recent years and is expected to continue so in the next. This is mainly owing to the advent of novel disruptive technologies based on AI [169] and advanced additive manufacturing [95,170,171].

## 5. Advanced Additive Manufacturing

**Figure 5:**
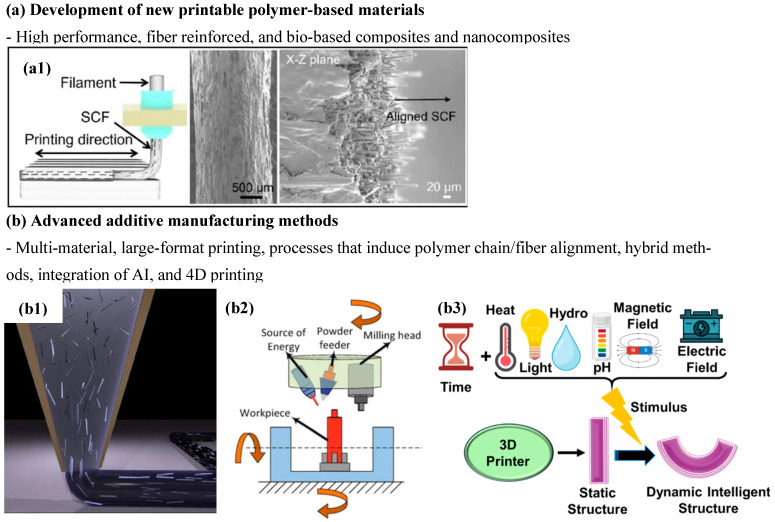
Main aspects of interest in AM: (**a**) development of new printable polymer-based materials—(**a1**) 3D printing of short carbon fiber-reinforced composites with controlled fiber orientation. (adapted from [198]. Springer Nature, 2024); and (**b**) advanced additive manufacturing methods—(**b1**) illustration of the alignment of fibers during the deposition of a polymer composite ink (adapted from [221]. Wiley Advanced, 2014), (**b2**) hybrid AM consisting of an integrated laser powder head direct energy deposition with multi-axis milling (adapted from [222]. Springer Nature, 2021), and (**b3**) representation of 4D printing incorporating time as dimension (adapted from [213]. Elsevier, 2025).

Since the advent and later extended use of additive manufacturing (AM), often called 3D printing, there has been a great interest in further extending the possibilities of the technique, since its beginning intended to facilitate the transition from design to real prototype with the ultimate desire of enabling the industrial production of real parts, especially since significant improvements in the reliability and raw material possibilities (including high-performance polymer-based materials or the already addressed bio-based composites) were attained [174,175], to the preparation of polymer-based components with enhanced mechanical performance and durability for highly demanding applications [176]. Two main aspects have been the focus of interest (Figure 5): the development of novel printable polymer-based materials (high-performance, fiber-reinforced, and bio-based composites and nanocomposites, normally used in FDM and continuous filament fabrication) and advanced 3D printing methods, including multi-material [177] and large-format printing [178], hybrid additive manufacturing, processes that induce polymer chain/fiber alignment or the integration of AI for optimization and quality control [179,180], and, more recently, 4D printing.

Notable recent breakthroughs, which are expected to continue in coming years, have dealt with the development of novel printable thermoplastic-based materials [181], mainly (nano)composites (for instance, reinforced with carbon nanotubes and/or graphene [182,183,184,185]), with enhanced printing abilities and resulting part strength (besides possible additional advantages such as thermal conduction), significantly decreasing material waste. Bio-based options, namely bio-based polymer composites, have also experienced a great deal of development, as it has been established as one of the priorities of AM techniques that materials and parts need to be sustainable and environmentally friendly [186,187,188]. In this sense, researchers have also considered the possibility of tailoring bio-based polymer blends for improved mechanical performance [189,190], as well as the already mentioned use of smart materials, more specifically SMPs [191,192,193].

One interesting trend in 3D printing is hybrid additive manufacturing. This basically combines, into one single process, an AM technique, such as powder bed fusion, with a secondary process, from traditional milling or drilling to welding. It uses a custom head machine tool capable of performing this dual task, removing or adding material when required [176,194]. This eliminates common defects of 3D parts such as pores and enables precise finishing of the part (considerably reducing post-processing time), two of the most negative aspects of 3D printing. Examples of hybrid AM include wire arc AM, which has been shown to enhance surface quality and part precision [195], laser metal deposition integration, laser-assisted methods such as LOMM or LAM, or techniques that use vibration. Nevertheless, there are still some important limitations to the extended industrial use of these techniques, owing to their complexity, comparatively higher cost (equipment and maintenance), longer production times, and more critical monitoring of the process [196,197].

Recent advancements in AM have introduced technical novelties in terms of controlling polymer chain and fiber alignment in printable composites. This is especially true for continuous fiber reinforced polymer composites [198,199,200]. The objective is to maximize mechanical performance through induced chain/fiber alignment. It also aims to solve one of the main problems of AM related to the intrinsic and unpredictable anisotropic characteristics of the parts, due to the common use of layer-by-layer manufacturing processes. Generally speaking, the degree of anisotropy may be controlled through several approaches. These include the printing material, such as adjusting the rheological characteristics by adding (nano)reinforcements [201,202,203,204,205] or blending low- and high-molecular-weight polymers [206]. It can also be managed via the process and printing parameters [207], which may generate the required shear forces on the material to promote molecular/fiber alignment [208]. Additionally, selecting proper post-processing techniques, such as thermal annealing [209], is effective. Vibration-assisted methods (such as the vibration-integrated auger extension system, VIAES) have recently been considered as a viable option to avoid fiber aggregation during printing and especially to effectively align the fibers in the printed part, leading to components with enhanced mechanical strength [210].

Only recently, significant experimental and computational technical advances have been made in terms of precisely detecting and controlling the orientation of polymer chains and fibers in polymer composites, critical for optimizing parts prepared by AM and ensuring product quality [211]. While common detection techniques have relied on direct imaging (TEM/SEM) or indirect measurement methods (such as in situ DRX characterization or ultrasonic measurements), as there is no individual technique that universally enables the assessment of all system orientation states, advancements have been made in combining more than one detection technique (multimodal approach), namely the high-resolution static images given by direct imaging with the real-time dynamic orientation monitoring during processing of the indirect methods. Research points in the direction of using integral methods that combine AI with surface/interface engineering and multiscale modeling, with the development of deep learning algorithms to decode complex data obtained from DRX/neutron or dielectric spectra for real-time assessment of the orientation during extrusion/injection molding, integration of sensors with multiscale molecular modeling, sensors based on metamaterials for mapping nanoscale orientation, or neural networks adapted to real-time acquired orientation data.

The integration of AI and ML has also been the subject of great interest. The conjunction of the two may optimize the mechanical performance and durability of the produced parts through a rational combination of raw materials (types and proportions) and AM processing and parameter selection, as well as automate post-processing [212]. AI, ML, and modeling in general are addressed in more detail in the following section. This is especially true when dealing with the characterization of complex mechanical behaviors, including parts produced by advanced additive manufacturing.

4D printing takes additive manufacturing a bit further, combining 3D printing and the use of smart materials, with the support of potent modeling and ML algorithms, resulting in complex parts that may evolve with time when in use without any computer aid, meaning that they may change shape or functionality when applying an external environmental stimulus, such as heat or light, enhancing part efficiency and durability [213,214,215,216]. Hence, 4D printing is becoming a promising technique in the development of what are known as “self-adaptive components” for a vast array of applications, from tissue regeneration to the construction sector [217,218,219]. This is especially true when considering the possibility of using continuous fiber-reinforced polymers. These are expected to enhance mechanical performance, enabling the use of printed parts in structural engineering applications. They can also counteract the limitations of polymers in terms of low/slow recovery stress. Additionally, they may add specific functionalities, such as electrical conductivity, for instance, for actuators. Or, as already mentioned, they enable tailored control of the distribution and orientation of fibers during processing, inducing or limiting mechanical anisotropy. All in all, this offers great potential in terms of design, manufacturing, and use of parts [220]. Challenges in the 4D printing of continuous fiber-reinforced composites include the possible use of hybrid fibers and/or previously functionalized fibers for improved interface compatibility; consideration of complex part structures, such as origami-like; the use of hybrid additive manufacturing and multi-material printing; and integration of advanced attributes, such as self-sensing or self-healing.

To wrap it up, additive manufacturing faces the following general overall challenges if it to be considered as a viable technique to the industrial production of advanced functional polymer-based parts with enhanced mechanical properties for structural applications: (1) high capital costs associated with AM equipment, certainly with respect to a larger scale, and the possible use of more expensive new experimental materials; (2) availability of new printing materials (types and amount), with durability limitations in functional components for some printable materials; (3) productivity, more specifically the slowness of several additive manufacturing technologies, which restrict widespread applications in various sectors (in this sense, previously mentioned hybrid methods could solve this problem, though they are still under development); (4) part inconsistency in many AM technologies due to raw material composition and storage variability; (5) post-processing required to achieve the desired quality, adding more time and cost to the overall manufacturing process, with the possible solution of automation; (6) limited scope for data preparation and design capabilities, with the possible solution of implementing a robust digital infrastructure that can efficiently handle several operational aspects; (7) limited methodology for material selection based on AM’s application; and (8) standardization, still under review and not fully implemented, limiting industrialization.

## 6. AI and Modeling

**Figure 6:**
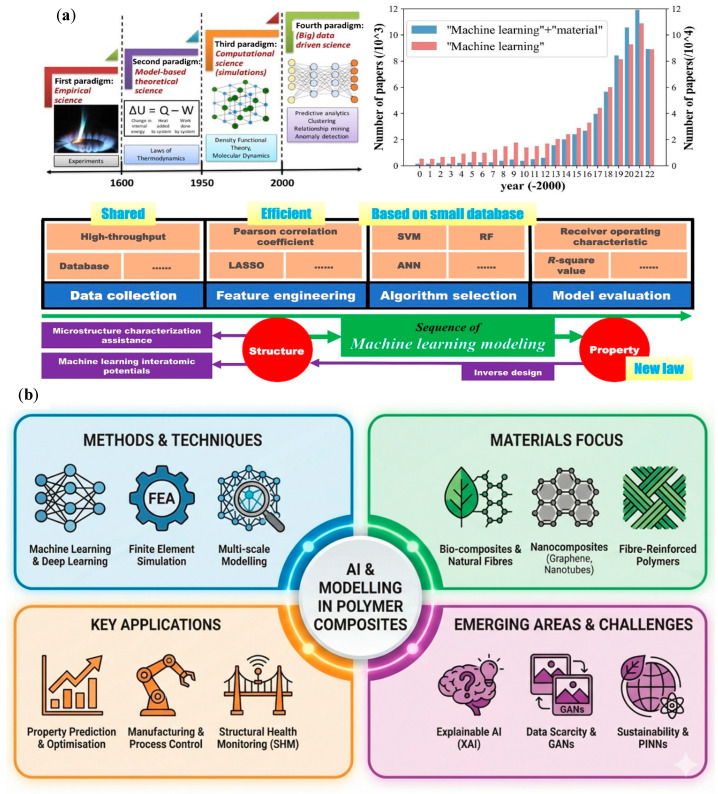

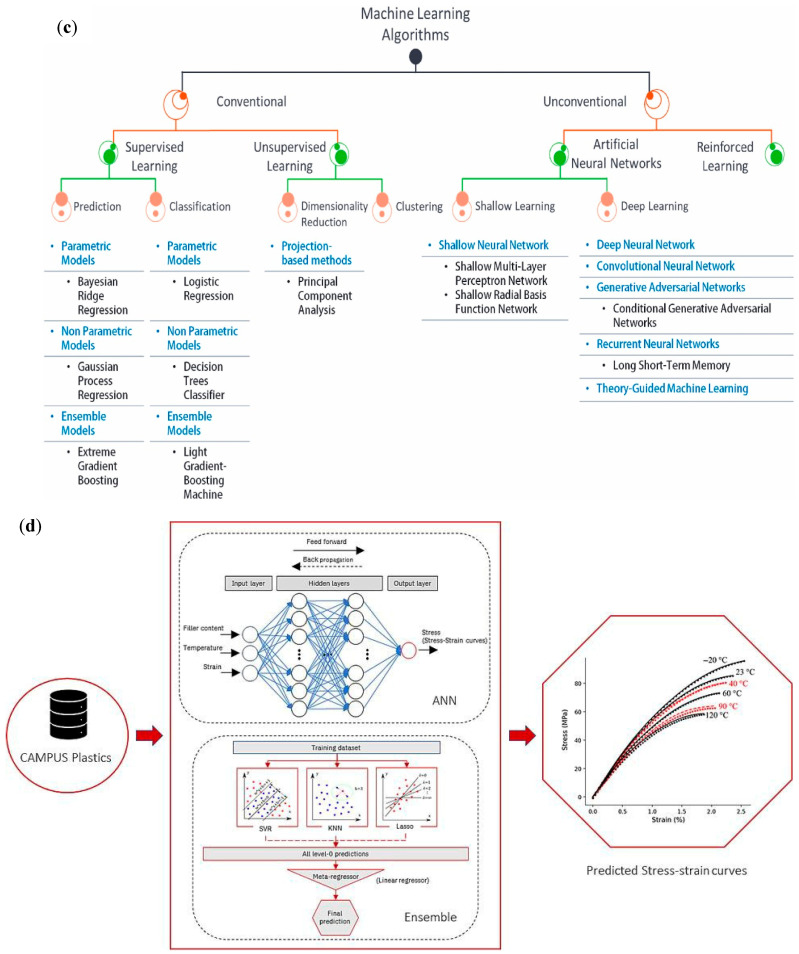
(**a**) Historical evolution and increasing research interest of ML (adapted from [223]. Wiley, 2023); (**b**) methods, materials, key applications, and challenges in the use of AI and modeling to polymer composites; (**c**) ML algorithms (adapted from [249]. Elsevier, 2024); and (**d**) example of an ML neural network with predicted stress–strain curves (adapted from [230]. Elsevier, 2023).

The integration of artificial intelligence (AI) and advanced modeling techniques has emerged as a transformative force in the field of polymer-based materials and composites. Often described as materials informatics [223], this paradigm shift is accelerating the discovery, characterization, and optimization of complex multiphase systems at an unprecedented rate (see Figure 6a). Leveraging data-driven approaches enables researchers to decipher intricate structure–property relationships that were previously beyond the orbit of traditional experimental methods (see the methods and techniques, materials, key applications, and challenges when using AI and modeling for polymer-based composites in Figure 6b).

One of the most prominent applications of AI in this field is predicting the mechanical properties of fiber-reinforced composites. The heterogeneity of these materials, which often include natural fibers and/or micro- or nanofillers, introduces non-linearities that challenge conventional analytical models. In this sense, Kibrete et al. [224] demonstrated the effectiveness of AI-based algorithms in accurately predicting the mechanical performance of composites, giving a valid alternative to expensive and time-consuming experimental testing. Similarly, Marrivada et al. [225] used machine learning (ML) techniques to predict the mechanical properties of triaxial-braided composites reinforced with graphene nanoplatelets, successfully assessing the influence of nanoparticle dispersion (nanoscale) on the material’s performance (macroscale).

Deep learning architectures and, more specifically, neural networks have demonstrated remarkable versatility in constitutive modeling (see ML algorithms shown in Figure 6c). In this sense, Maia et al. [226] used physically recurrent neural networks (PRNNs) for modeling heterogeneous materials, directly introducing physical laws into data-driven metamodels to guarantee thermodynamic consistency. This approach is essential for materials that exhibit complex, history-dependent behaviors, inherent to the plastic-like deformation and ductile fracture of most polymers. In the context of bio-composites, Wang et al. [227] combined finite element simulation with ML to elucidate the tensile performance mechanisms of eco-friendly bamboo fiber-reinforced, palm oil-based resin composites. This highlights how hybrid approaches can reveal possible synergistic effects between the different constituents present in composites.

Specific mechanical behaviors, such as tribological performance and viscoelasticity, are also being targeted. For example, Teli et al. [228] successfully used artificial neural networks (ANNs) to study the tribological behavior of hemp particulate-filled epoxy composites, optimizing hemp content to improve wear resistance. Furthermore, Chen et al. [229] examined the viscoelasticity of bio-based composites, developing models that accurately predict time-dependent deformation in PLA and regenerated cellulose fiber systems. These studies demonstrate the ability of ML to solve multi-parameter optimization problems inherent to the complexity of multiphase systems.

AI is pivotal in accelerating the design of new materials beyond property prediction. Esmaeili and Rizvi [230] proposed an accelerated strategy involving ensemble learning approaches for characterizing the mechanical properties of polymer composites (see Figure 6d). This significantly reduces the experimental dataset required to achieve high predictive accuracy. This “small data” capability is particularly valuable in Materials Science, where generating large datasets is resource intensive. Wang et al. [231] reviewed sequential deep feed-forward neural networks (DFF) and showed their effectiveness in designing the layout of composites.

The role of AI in the management of composite materials extends to their entire lifecycle through intelligent structural health monitoring (SHM). A review from 2024 by Azad et al. [232] emphasized the importance of integrating machine, deep and transfer learning methods into SHM systems to permit instantaneous detection and, damage prediction in composite structures, crucial in applications where part integrity is paramount, such as aerospace or automotive sectors.

Recent developments in 2025 have focused in multiscale modeling, bridging the gap between microscopic/nanoscale interactions and macroscopic performance. Yang et al. [233] investigated the impact of microscale fiber uncertainties on the mechanical behavior of natural/synthetic hybrid fiber composites, shedding light on how stochastic variations at the fiber level lead to failure at the structural level. Furthermore, Zhao et al. [234] used multiscale simulations to design synergistic enhancements in basalt fiber-reinforced composites with nanotube and graphene bridging structures, demonstrating the effectiveness of computational tools in designing hierarchical reinforcements.

Concerning green composites, Al-Oqla et al. [235] developed an adaptive neuro-fuzzy inference system (ANFIS) to predict the mechanical properties of several types of cellulose fibers, interestingly addressing the inherent variability of natural fibers, generating reliable property data and hence making them more suitable for industrial applications. Liu et al. [236] also advanced on the understanding of interfacial mechanisms by conducting a multiscale study on the impact of SiO_2_ nano-interphases. This study clarified how nano-modifications influence water absorption and mechanical retention.

Further advances in manufacturing have focused on making AI models more interpretable to optimize processing parameters. Ma et al. [237] used a new explainable machine learning (XML) method to analyze how the microstructure affects the machinability of natural fiber-reinforced plastic composites. By making the “black box” of ML transparent, they provided actionable insights into how fiber orientation and distribution directly impact machining quality, a critical step for the industrial-scale production of these materials. Concurrently, significant progress has been made in modeling damage evolution. Han et al. [238] developed an ANN-based concurrent multiscale damage evolution model for hierarchical fiber-reinforced composites that accurately tracks progressive failure mechanisms from the nanoscale to the macroscale. Building on this, Ghane et al. [239] proposed a multiscale deep learning model for predicting the elastic properties of woven composites, which can effectively handle the geometric complexities of textile reinforcements.

The pursuit of physical consistency in modeling has also given rise to new frameworks that combine statistical mechanics with machine learning. In terms of micromechanics, Königsberger et al. [240] advanced the upscaling of stiffness and elastic limits in plant fiber-reinforced composites by transitioning from cellulose nanofibrils to technical fibers. Likewise, Li et al. [241] examined the viscoelastic properties of these materials using multiscale micromechanics modeling to predict time-dependent responses in natural plant fibers, crucial for predicting the materials’ long-term durability.

As we move through 2025, the consolidation of these techniques is evident in the growing number of comprehensive reviews that address the intersection of AI and polymer composites. For example, Mulenga et al. [242] and Alagulakshmi et al. [243] have provided extensive overviews of ML methods in natural fiber and fiber-reinforced polymer composites, respectively, showing that data-driven design methods are becoming standard. Furthermore, Uddin et al. [244] showed the role of intelligent algorithms in overcoming the characterization variability of biodegradable polymers inherent to their brittleness by applying AI to PLA composites. These studies suggest a future where “smart” modeling can predict performance and actively guide the development of sustainable high-performance composites.

However, significant challenges still remain. To begin with, the lack of reliable experimental data, which has prompted the use of Generative Adversarial Networks (GANs) to generate condensed accurate databases [245]. The interpretability of these models is being addressed by Explainable AI (XAI) techniques, such as Shapley Additive Explanations (SHAP), which directly link predictions to the underlying mechanics [246,247]. Finally, the environmental sensitivity of natural composites requires the adoption of physics-informed neural networks (PINNs) [248].

**Table 1:** Strategies and main outcomes and challenges regarding the development of polymer-based (nano)composites with enhanced mechanical properties.

Strategy	Main Outcomes	Main Challenges
Advanced composites based on high-temperature polymers and blends	- Development of thermoplastic materials with enhanced mechanical performance [3,4] - New AM techniques - Use of block copolymers for selective localization of nanoparticles [7,8,9,10,11]	- Issues about the long-term viability of 3D printed parts - AI and ML integration (use of reliable data) - Use of viable sustainable materials - Issues on how to properly assess the dynamic real-time behavior of these systems
Advanced polymer-based nanocomposites	- Significant mechanical improvements when adding nanoreinforcements [12,13,14,15,16,17,18,19] - Enhanced functional properties when adding carbon nanofillers [12,13,14,15,16,17,18,19,20,21,22,23] - Further and more efficient mechanical/functional characteristics when adding nanohybrids [27,28,29,30,31] or multiscale reinforcements [48,49,50] - Bio-based nanocomposites with improved mechanical properties based on biopolymers and nanocellulose [54,57,58,59,60,61,62,63,64,65,66,67,68,69,70]	- Limited material availability - Problems in guaranteeing a homogeneous distribution/dispersion of the nanoparticles - Limited long-term mechanical and thermal stabilities - Development of reliable sustainable materials (bio-based) - AI and ML integration for material design
Porous polymer-based structures	- Development of lightweight components with improved specific properties [71,72,73,74,75,76,77,78,79] - Functionally graded porous structures adapted to part requirements [87,88,89,90] - The use of AM has enabled the more versatile creation of graded structures [91,92,93,94,95,96,97]	- Creation of design guidelines using AI and ML - Development of new printable materials, preferably sustainable - Development of large-scale processing methods
Interface engineering	- Identification, prediction, and control of the correlations between the interface(s) across the different materials’ scales and the multifunctional properties of the composites [99,100,101,106,107,108]	- How to effectively surface modify the nanosized phase(s) without affecting their intrinsic properties - How to incorporate the surface-modified nanoreinforcements into the matrix - Scale-up to high-volume processes - How to accurately evaluate the interface properties in these multiphase systems
Bio-based polymer (nano)composites	- Recent mechanical improvements when combining bio-based recyclable polymers with biofibers and/or nanocellulose [121,122,123] - Development of new thermoplastic-based “green” formulations [148,150,151,152]	- Traceability and control of the raw materials - Raw material cost variability - Possible problems when incorporating nanofillers, especially bio-based such as nanocellulose into bio-based polymers - Stability of the composites - Development of new processes, especially AM-based ones, in order to effectively transfer from the lab to the industry - How to transfer/integrate all these possibilities into practical applications
Multifunctional polymer-based materials	- Lifespan extension and monitoring through multifunctionality (smart materials [156,157,159,160,161]) - Development of metamaterials [158,168]	- The use of mechanical metamaterials has not reached its technological expectations
Advanced additive manufacturing	- Use of AM to prepare parts with enhanced mechanical performance and durability [176] - Development of new continuously-reinforced printable materials [181,182,183,184,185] - Advanced 3D printing: hybrid methods and 4D printing [177,178,179,180] - Use of fully bio-based systems [186,187,188,195] - AM methods that control fiber orientation [198,199,200,201,202,203,204,205,206,207,208,209,210] - AI and ML integration and automate post-processing [212] - Nascent 4D printing for the creation of self-adaptive parts [217,218,219]	- Higher complexity, comparatively higher cost (equipment and maintenance), and longer production times - More critical monitoring of hybrid processes - Use of hybrid fibers in 4D printing - Consideration of complex printed structures - Integration of self-sensing and healing - Limited methodology for material selection
AI and modeling	- Prediction of the mechanical properties of fiber-reinforced composites [224,225,226,227,228,229] - Design of new materials beyond property prediction [230,231] - Multiscale modeling [233,234] - Significant progresses in damage evolution [239]	- Lack of reliable experimental data - Necessity to use complex ML methods based on deep learning neural networks

**Table 2:** Strategies, examples of materials, processing methods, mechanical enhancements, and end-use applications of polymer-based (nano)composites.

Strategy	Material	Processing Method	Mechanical Enhancement	End-Use/Field of Application
Advanced composites based on high-temperature thermoplastics and blends	PAEK/CF [4]	Compression molding	- Tensile strength: 590.2 MPa (>9% increase vs. untreated PAEK/CF)	Structural, aerospace, and nuclear applications
	PAEK/CF [4]	Compression molding	- Elastic modulus: 22.8 GPa - Tensile strength: 680 MPa (>20% increase post-plasma treatment)	
	PAEK/CF/CNT or GnP coated [4]	Compression molding	- G_IC_ around 45 kJ/m^2^ (almost 300% increase) - Storage modulus at room temperature: 12.5 GPa (>300% increase)	
	PAEK/PBO [4]	Compression molding	- Tensile strength: 720.0 MPa (27% increase post plasma treatment)	
	PEEK/CF [4]	Vacuum hot pressing	- Hardness: 90 HRM (>150% increase) - Wear rate: 2.13 × 10^−6^ mm^3^ (N·m)^−1^ (>1 order of magnitude reduction) - Friction coefficient: 0.10 (60% reduction)	Structural and medical applications
	PEKK/CF [4]	3D printed and 3D printed + compression pressed	- Flexural modulus: 8.2 GPa - Flexural strength: 257.2 MPa (>130% increase)	
	PSPE-PB-PS block copolymer + clay nanoparticles [10]	Melt mixing and compression molding	- Young’s modulus: 52.5 MPa (almost 200% increase)	Sensors
Advanced polymer-based nanocomposites	PC/CNT/CF fiber composites (multiscale) [4]	(1) CNT dispersion in chloroform and PC dissolution; (2) impregnation of CNT/PC/chloroform solution into woven CF and evaporation	- Storage modulus: 39 GPa (>20% increase vs. PC/CF) - Impact energy: 5.3 J (200% increase vs. PC/CF)	Structural, aerospace, and nuclear applications
	PES/short CF/GO (multiscale) [50]	Injection molding	- Young’s modulus: 8 GPa (↑ 30% increase vs. PES/CF) - Tensile strength: 120 MPa (↑ 12% increase vs. PES/CF)	
	PDMS/MXene/CNT [21]	Layer-by-layer deposition	- Young’s modulus 15 times higher than PDMS - Less than 12% strain vs. 200% strain for PDMS	Sensors and actuators
	PI/CNT-GO nanohybrids [28]	Solution casting	- Tensile strength: 162.0 MPa (118% increase) - Young’s modulus: 4.0 GPa (almost 100% increase) - Toughness: 30.2 MPa (138% increase)	
	TPU/CNT-GnP nanohybrids [31]	Printing on a fabric substrate	- High strain sensitivity: gauge factor > 210 - Broad working sensing range: 112% strain	
	MXene/nanocellulose film [64]	Vacuum filtration-induced self-assembly	- Tensile strength: 128.2 MPa - Strain at break: 5.3%	
	PEI/CF/cellulose nanocrystals [69]	Layer-by-layer assembly technique	Interfacial shear strength (IFSS): 77.65 MPa (>138% increase vs. PEI/CF)	
	MXene/cellulose nanofibers film [64]	Vacuum filtration-induced self-assembly	- Mechanical strength: 112.5 MPa - Toughness: 2.7 MJ⋅m^−3^ (alternating multilayer film)	EMI shielding
Porous polymer-based structures	PUR/casein [71]	One-step free foaming method	Compressive strength: 0.144 MPa (>100% increase)	Mechanical energy dissipation and conversion
	PI [71]	Microwave-assisted foaming and post-curing	Compression recovery rate close to 100%	
	PU [72]	Microcellular one-step free foaming method	Compressive strength at 10% strain: 0.55 MPa (>5000% increase; comparable to commercial rigid foams)	
	PU/nanocellulose [78]	One-step free foaming method	Enhanced core shear stress (90%) and core shear strength (55%) in sandwich panels	Structural lightweight construction systems
	Functionally graded porous nanocomposites [87]	Direct templating, in situ and melt intercalation, and solvent methods	Enhanced compressive and tensile stresses, energy absorption, and stress distribution endurance	
Multifunctional polymer-based materials	PU/cellulose crystals [156]	Suspension casting	- Improved stiffness while keeping shape memory performance	Sensors, controllable devices, and adaptive and deployable structures
	PLA/TPU/HA blends [159]	Solution mixing and pouring into mold	- Improved mechanical strength (36 MPa, 66% increase) while keeping shape memory performance (>6% strain at break)	Biomedical applications
Advanced additive manufacturing	PLA/GnP [176]	Fused deposition modeling (FDM)	- Tensile strength: 96.5 MPa (after annealing) (>120% increase) - Flexural strength: 101.8 MPa (after annealing) (almost 50% increase)	Biodegradable parts for automotive, aerospace, electronics, and medical sectors
	PLA/CNT [185]	Fused deposition modeling (FDM)	- Elastic modulus: 5.7 GPa - Maximum stress: 1087 MPa - (26% increase)	
	PLA/CF [199]	Fused deposition modeling (FDM)	- Tensile strength: 1090.2 MPa - Flexural strength: 566.8 MPa - Shear strength: 11.4 MPa	
	PA1212 short CF [202]	3D laser sintering	- Tensile strength: 80 MPa - (67% increase) - Young’s modulus: 5.6 GPa - (>200% increase)	
	PP/nanocrystalline cellulose [208]	One-step compounding	- Tensile strength: 34.8 MPa - Flexural strength: 57.3 MPa	
	Pluronic F127 dimethacrylate/PAM [176]	Direct Ink Writing (DIW)	- Prepared hydrogels endured compression up to 90% without breakage and reverted to their original state - Improved modulus (0.931 MPa), 85% of equivalent molded hydrogel	Biomedical applications (in vitro blood vessel simulation)
	SMPU/CNT/HNT [213]	4D printing	- Tensile strength: 29.0 MPa - (almost 200% increase) - Flexural strength: 52.3 MPa - (almost 300% increase) - Shape recovery time: 180 s - (40% reduction)	Biomedical devices, smart textiles, aerospace components, and actuators

## Data Availability

No new data were created or analyzed in this study. Data sharing is not applicable to this article.
